# Glycogen Storage Disease type 1a – a secondary cause for hyperlipidemia: report of five cases

**DOI:** 10.1186/2251-6581-12-25

**Published:** 2013-06-06

**Authors:** Patrícia Margarida Serra Carvalho, Nuno José Marques Mendes Silva, Patrícia Glória Dinis Dias, João Filipe Cordeiro Porto, Lèlita Conceição Santos, José Manuel Nascimento Costa

**Affiliations:** 1 Internal Medicine Ward, Coimbra University Hospital, Av. Bissaya Barreto e Praceta Prof, Mota Pinto, 3000-075 Coimbra Portugal; 2 Serviço de Medicina, Enfermaria D, Centro Hospitalar e, Universitário de Coimbra, Avenida Bissaya Barreto e Praceta Prof, Mota Pinto, 3000-075 Coimbra, Portugal

**Keywords:** Glycogen storage disease type Ia, Glucose-6-phosphatase-α, Hypoglycemia, Hyperlactacidemia, Hyperlipidemia, Hyperuricemia

## Abstract

**Background and aims:**

Glycogen storage disease type Ia (GSD Ia) is a rare metabolic disorder, caused by deficient activity of glucose-6-phosphatase-α. It produces fasting induced hypoglycemia and hepatomegaly, usually manifested in the first semester of life. Besides, it is also associated with growth delay, anemia, platelet dysfunction, osteopenia and sometimes osteoporosis. Hyperlipidemia and hyperuricemia are almost always present and hepatocellular adenomas and renal dysfunction frequent late complications.

**Methods:**

The authors present a report of five adult patients with GSD Ia followed in internal medicine appointments and subspecialties.

**Results:**

Four out of five patients were diagnosed in the first 6 months of life, while the other one was diagnosed in adult life after the discovery of hepatocellular adenomas. In two cases genetic tests were performed, being identified the missense mutation R83C in one, and the mutation IVS4-3C > G in the intron 4 of glucose-6-phosphatase gene, not previously described, in the other. Growth retardation was present in 3 patients, and all of them had anemia, increased bleeding tendency and hepatocellular adenomas; osteopenia/osteoporosis was present in three cases. All but one patient had marked hyperlipidemia and hyperuricemia, with evidence of endothelial dysfunction in one case and of brain damage with refractory epilepsy in another case. Proteinuria was present in two cases and end-stage renal disease in another case. There was a great variability in the dietary measures; in one case, liver transplantation was performed, with correction of the metabolic derangements.

**Conclusions:**

Hyperlipidemia is almost always present and only partially responds to dietary and drug therapy; liver transplantation is the only definitive solution. Although its association with premature atherosclerosis is rare, there have been reports of endothelial dysfunction, raising the possibility for increased cardiovascular risk in this group of patients. Being a rare disease, no single metabolic center has experience with large numbers of patients and the recommendations are based on clinical experience more than large scale studies.

## Background

Glycogen storage disease type I (GSD I) comprises a group of relatively rare autosomal recessive inherited metabolic disorders. It is caused by deficient activity of glucose-6-phosphatase system (G6Pase), an enzyme complex that plays a major role in both glycogenolisis and gluconeogenesis [1]. This complex is required for the hydrolysis of glucose-6-phosphate (G6P) into glucose and inorganic phosphate [2].

The annual incidence is about 1/100000 births [1–3] and the carrier frequency of 1 in 150 [4], being the GSD type Ia (McKusick, #232200) the more frequent type, accounting for 80% of the GSD I patients. It is caused by mutations in the *G6PC*, located on chromosome 17q21, which encodes the catalytic subunit of glucose-6-phosphatase-α (G6Pase-α), whose expression is limited to the liver, kidney and intestinal mucosa [2, 3]; more than 85 mutations have been identified [5, 6].

The disturbed glucose homeostasis associated with the inability to breakdown glycogen stores [7] makes patients prone to fast induced hypoglycemia, with secondary metabolic derangements: hyperlactacidemia, hyperlipidemia and hyperuricemia. Another common manifestation is a protruded abdomen due to marked hepatomegaly; patients usually present before 1 year and have a round “doll like” face, growth and psychomotor delay and late onset of puberty, subclinical muscle weakness, osteopenia, platelet dysfunction, with bleeding tendency, anemia and intermittent diarrhea; in women polycystic ovaries are usually found [8].

The diagnosis can be based on clinical and biochemical findings, functional tests, enzyme assays of liver biopsy tissue and mutation analysis; currently, the increased knowledge of the genetic basis of GSD I makes the mutation analysis the method of choice for confirming the diagnosis [1, 2, 9].

A poor metabolic control may lead to long term complications, namely development of hepatocellular adenomas (HCA), renal dysfunction, gout and nephrocalcinosis associated with hyperuricemia, and increased risk of pancreatitis, related to severe hypertrigliceridemia [1, 2].

The aim of the treatment, which is mainly dietary, is to maintain a nearly euglycemic state to prevent secondary metabolic disturbances, neurologic involvement and assure normal growth [2, 9].

The rarity of this condition implies that no single metabolic centre has experience with large number of patients. Being the diagnosis made usually in childhood, the follow up is done in pediatric centers. As the life expectancy has improved considerably, these patients reach adult life and need to maintain follow up of the complications associated with the natural history of the disease. One of the manifestations almost always present is severe hyperlipidemia, rarely associated with premature atherosclerosis, although some studies report an increased cardiovascular risk [10]. GSD I affects many organ systems (renal, gastrointestinal, hematological, bone, cardiovascular, etc.) and a multidisciplinary approach, including nutritional support, is required to ameliorate the complications frequently present. It is important to be familiarized with the disease.

The authors present the report of five adult patients with GSD type Ia followed in internal medicine appointments and subspecialties [1, 9].

## Case presentation

### Case 1

The first case refers to a 33 year-old man, diagnosed with GSD type Ia at five months of age. Three days after an uncomplicated birth from second degree relative parents, he had a seizure related to severe hypoglycemia (1,27 mmol/L), which recurred at four and five months. By this time, a protruded abdomen associated with enlarged liver was noted (13 cm bellow costal margin), with elevated liver enzymes (six times the upper limit of normal), lactic acidosis e hyperuricemia (523 mcmol/L, with normal range between 118 and 356 mcmol/L).

To confirm the diagnosis two functional tests were performed: the injection of glucagon caused an elevation of lactate and uric acid and a decrease in serum glucose, and the administration of galactose didn’t had any effect on glycemia, proving the absence of G6Pase-α, which was confirmed by liver biopsy four years later, also showing increased glycogen storage in the liver. The mutation analysis, performed at the age of 20 revealed a homozygous mutation (IVS4-3C > G) in the intron 4 of the *G6PC*, also present in both is parents as a heterozygous trait. The family history of our proband was marked by the death in the neonatal period of two older sisters, the first with hepatomegaly and the second with seizures and suspected congenital heart disease.

During childhood he had poor metabolic control, with frequent episodes of hypoglycemia and acidosis, epistaxis and muscle weakness, which improved only partially after he was started on uncooked cornstarch (UCCS) at the age of 4 years and continuous nocturnal gastric drip feeding (CNGDF) at the age of 5, replaced when 10 years with two nocturnal administrations of UCCS, maintained in adulthood.

Because he had short stature and delayed puberty he started testosterone supplementation at 15 years. A cognitive impairment was also noted (global IQ of 67). He has now a height of 1,51 m and a weight of 56 Kg (body mass index of 25,6 Kg/m^2^).

The unsatisfactory metabolic control led to hyperuricemia, under therapy with alopurinol 300 mg once daily since he was 9, and hyperlipidemia since the age of 20, with total cholesterol over 10 mmol/L and triglycerides over 21 mmol/L, with raised apolipoprotein B100 and Apolipoprotein B100/Apolipoprotein A1 ratio. He was started on fibrates at 25, with good response (triglycerides bellow 3 mmol/L), but this medication was withdrawn after he developed myalgia and muscle weakness related with rhabdomyolysis (creatine kinase over 29000 U/L). Despite this severe hyperlipidemia, there are no signs of atherosclerotic lesions, with normal carotid ultrasound and exercise ECG.

He has sustained hepatomegaly and steatosis since infancy; after 20 years he developed multiple HCA, the largest over 5 cm, whose histology revealed focal nodular hyperplasia. Until now there are no signs of malignant transformation.

Since childhood he had frequent hospital admissions for upper gastrointestinal bleeding, being diagnosed with duodenitis at 12 years old. Six years after he was submitted to an exploratory laparotomy for the same reason, without identification of the source of the bleeding. Later, an esophagogastroduodenoscopy revealed a raised formation in the duodenal bulbous, which was biopsied and showed adenomatous changes. Recently, he had another episode of upper gastrointestinal bleeding: a well differentiated neuroendocrine tumor of the first portion of the duodenum, with lymph node metastasis was diagnosed after exploratory laparotomy.

Our patient also suffers from renal dysfunction. Besides an episode of acute glomerulonephritis without identifiable cause at 14 years, he has proteinuria since the age of 6, under therapy with captopril 25 mg twice daily started when 17 years old. He has neither nephrocalcinosis nor urolithiasis and is under therapy with citrate supplementation since childhood. Notwithstanding being under therapy, he developed nephrotic range proteinuria (over 5 g/day) which evolved recently to end-stage renal disease, needing to start hemodialysis.

Furthermore he also has low bone mineral density diagnosed at 18 years (*Tscore* of −2,9 at lumbar spine and *Tscore* of −3,6 at the femur neck, *Z score* of −3,21) and chronic anemia since infancy, with need for frequent supplementation with iron and/or folic acid. He had two episodes of facial palsy, the first one at 15 years.

In the present moment he is under therapy with iron, folic acid, calcium and vitamin D, angiotensin converting enzyme inhibitor, angiotensin receptor blocker, alopurinol, statin and darbopoetin.

### Case 2

The second case refers to a 51 year-old man, diagnosed with GSD type Ia in adulthood (30 years old), after the detection four years earlier of hepatomegaly with multiple adenomas, the largest with 11 cm of diameter. The diagnosis was confirmed by deficiency of G6Pase-α activity in liver biopsy tissue and evidence of hyperlactacidemia. There is no information about whether genetic tests were performed.

Besides frequent epistaxis and an episode of enteritis, he had a normal childhood, without symptomatic hypoglycemic episodes (median fast glucose determinations of 3,3 mmol/L) as long he maintained frequent meals with carbohydrate rich food.

When 15 years old, short stature and delayed puberty were noticed and he was started, at first, on human chorionic gonadotropin, and then testosterone supplementation. Three years later, he was admitted with hyperuricemia (over 700 mcmol/L) associated gouty arthritis and he was started on alopurinol 300 mg once daily. His blood chemistry revealed hyperlactacidemia and hyperlipidemia with total cholesterol over 7,7 mmol/L and triglyceride over 11,2 mmol/L. Apolipoprotein B100 and Apolipoprotein B100/Apolipoprotein A1 ratio were also elevated. He was started on ciprofibrate 100 mg once daily at 36 years, with poor response, replaced by fenofibrate 267 mg once daily combined with nicotinic acid 1 g twice daily, with good response (total cholesterol over 8 mmol/L and triglycerides over 4,8 mmol/L). The carotid ultrasound revealed atherosclerotic plaques, without hemodynamically significant stenosis and the electrocardiogram was suggestive of antero-lateral ischemia, not confirmed on myocardial perfusion scan. At 47 years old, omega-3-acid ethyl ester 1 g twice daily was introduced, in association with fenofibrate and nicotinic acid, with better response (total cholesterol below 5,5 mmol/L and triglycerides below 2,5 mmol/L).

Moreover, he had iron deficiency anemia known from 26 years old, refractory to iron supplementation. During follow up low bone mineral density was noticed (*Tscore* of – 3,3 at the lumbar spine and *Tscore* of −1,1 at the femur neck, *Z score* not available), with significant gain of bone mineral density after 3 years of therapy with biphosphonates. Important proteinuria was also noted (over 500 mg/24 hours), with slight elevation of creatinine (116 mcmol/L), which warranted a nephrologist referral. No urolithiasis was present, although he referred past history of renal colic. Gouty arthritis crisis occurred monthly and high blood pressure was diagnosed, with the need for antihypertensive therapy.

About his family history, he is the oldest of three brothers (the youngest with 47 and 41 years old, respectively) with the same diagnosis, the former with end-stage renal disease (focal and segmental glomerulosclerosis), under hemodialysis. In respect to offspring, he has a healthy daughter.

Furthermore short stature (height of 1,56 m, weight of 54 Kg and body mass index of 22 Kg/m^2^) and hepatomegaly, his physical exam is unremarkable. No cognitive dysfunction is noted.

After being diagnosed, he was started on UCCS, interrupted for bad compliance.

He is currently under therapy with losartan, acetylsalicylic acid, iron, folic acid, alopurinol, fenofibrate, omega-3-acid ethyl esters, nicotinic acid plus laropiprant and alendronic acid plus colecalciferol.

### Case 3

The third case refers to a 28 year-old woman diagnosed with glycogen storage disease type Ia in the neonatal period. She presented from birth with seizures and severe hypoglycemia; parenteral nutrition was instituted at first, then continuous enteric nutrition for a period of time, followed by continuous enteric nutrition during the night, replaced by frequent meals with maltodextrin and uncooked cornstarch as she grew older. Enzymatic studies performed in the liver tissue biopsy were suggestive of the disease, and a homozygous R83C mutation in the exon 2 of *G6PC* was identified.

Since childhood she was frequently admitted with seizures and gastrointestinal and upper airway infections.

She has hepatomegaly with multiple hepatocelullar adenomas, the largest with 4 cm of diameter, first diagnosed at 16 years old. She also has low bone mineral density (*Tscore* of −1,3 at the lumbar spine and *Tscore* −0,69 at the femur neck, with a *Z score* of – 0,6), sideropenic anemia and polycystic ovaries.

Unlike other cases, she has hypouricemia (71 mcmol/L) and a normal lipid profile, with total cholesterol below 5,5 mmol/L and triglycerides below 1,5 mmol/L; her HDL-cholesterol is raised (2 mmol/L). She also has an elevated Apolipoprotein A1, with normal Apolipoprotein B100 and Apolipoprotein B100/Apolipoprotein A1 ratio. There are no signs of renal involvement, namely proteinuria.

Short stature is a feature present in this patient (height of 1,5 m, weight of 60 Kg, and body mass index of 26, 7 Kg/m2) and during follow up there has been a weight gain associated with accumulation of fat in the subcutaneous tissue (lipomatosis) in connection with an enriched carbohydrate diet and frequent administrations of UCCS to prevent hypoglycemia. As in our first case, she has cognitive dysfunction, with a global IQ of 71.

Furthermore, she suffers from a refractory form of epilepsy, with almost daily crisis, not always related to hypoglycemic episodes. The electroencephalogram revealed interictal epileptic discharges in the right frontal region and the magnetic resonance imaging showed subcortical gliosis, right parieto-occipital atrophy, with enhanced sulcus and smaller right hemisphere.

She is now under therapy with levetiracetam 1000 mg twice daily, oxcarbamazepine 600 mg three times daily, clobazam 20 mg twice daily, sertraline 50 mg once daily, folic acid once daily, ferrous sulfate once daily and omeprazol once daily.

### Case 4

The forth case refers to a 36 year-old woman, presenting with vomiting, weight loss, protruding abdomen and hypoglycemic seizure when 2 months old. She had been previously admitted in the first week of life with vomiting. Epistaxis was frequent.

GSD type Ia was confirmed when 3 years old by liver biopsy (absence of G6Pase-α activity). There is no information whether genetic tests were performed.

HCA were identified very early, when 11 years old, and by this time she began continuous nocturnal gastric drip feeding, replaced by frequent meals and UCCS when 21 years-old.

Besides fasting hypoglycemia, she also had a mild hyperlipidemia (total cholesterol over 7,2 mmol/L; triglycerides over 3,4 mmol/L), with low HDL-cholesterol and raised Apolipoprotein B 100 and Apolipoprotein B100/Apolipoprotein A1 ratio. Hyperuricemia (uric acid over 550 mcmol/L) and mild normocytic anemia were also present.

Her renal function is normal, without significant proteinuria.

She has normal mineral bone density (*Tscore* of 0,6 at the lumbar spine and *Tscore* of 0,1 at the femur neck, with a *Z score* of 0,6 and 0,2, respectively) and irregular menstruation cycles. She has normal stature (height of 1,62 m, weight of 65 Kg, and body mass index of 24, 7 Kg/m2) and a normal cognitive function, with a college degree.

When she was 27 years old, having multiple HCA, the largest with 6 cm of diameter, she had liver transplantation. The anathomopathology revealed portal fibrosis, steatosis and multiple HCA and epithelioid granulomas. After liver transplantation her hypoglycemic crises, hyperlipidemia and hyperuricemia were corrected, without the need for pharmacological intervention.

Later on (2 years after transplantation) she became pregnant and delivered a healthy boy (38 weeks of gestation). During pregnancy there was a raise in liver enzymes and the liver biopsy revealed moderate late acute cellular rejection of the liver graft, with the need for corticotherapy and adjustment of the immunosuppressive therapy.

During follow up depression was noted, with the need for anti-depressant medication. She is now under immunosuppressors.

### Case 5

The fifth case refers to a 29 year-old man, sib to the previous patient, diagnosed with GSD Ia when 2 months old, as he had hypoglycemia, hyperlactacidemia and hepatomegaly, in association with a suggestive family history. His parents weren’t consanguineous. When 3 months, he started dietary treatment with frequent meals (every 3 hours), including 2 meals in the nocturnal period. In spite of that, he developed hyperlipidemia (total cholesterol over 4 mmol/L, triglycerides over 7 mmol/L), hyperuricemia (uric acid over 500 mcmol/L) and growth failure, and so, he started continuous nocturnal nasogastric drip feeding when he was 7 years, with recovery of normal growth. This dietary treatment was replaced with frequent meals and UCCS when 14 years old. His hyperlipidemia and hyperuricemia weren’t corrected with the dietary treatment, with the need for introduction of gemphibrozile and alopurinol when 19.

During adolescence, he had bad dietary treatment compliance, and he maintained marked hepatomegaly and steatosis, being diagnoses with multiple HCA when 19, the largest with 6 cm in diameter.

Besides, he has mild normocytic anemia and occasional epistaxis. He has now a height of 1,77 m, weight of 62 Kg and a body mass index of 19,7 Kg/m^2^. He maintains hyperlipidemia (total cholesterol over 5 mmol/L, triglycerides over 11 mmol/L), low HDL-cholesterol and hyperuricemia. He also developed proteinuria (400 mg/24 hours).

Table 1 resume the main findings of our five case reports.

**Table 1:** **Characteristics of the five GSD la patients**

Case	Age of diagnosis	Presentation (age)	Genetic tests	Hyperlipid emia	Hyperuric emia	Osteopenia/osteoporosis	Anemia	Platelet dysfunction	Growth delay/cognitive dysfunction	HCA (age of diagnosis)	Renal involvement	Therapy	Other features
33 y ♂	5 mo	Hypoglycemic seizures (3 days)	IVS4-3C > G	+++	+++	Osteoporosis	+	Epistaxis Upper gastrointestinal bleeding	+/+	+ (20 y)	Nephrotic range proteinuria	UCCS start for y → CNGDF from 5 to 10	Neuroendocrin tumor of duodenal wall
		Hepatomegal y (5 mo)									End-stage renal disease	y → UCCS	
51 y ♂	30 y	HCA (growth failure and epistaxis during childhood)	Not performed	+++	+++ (gouty arthritis)	Osteoporosis	+	Epistaxis	+/-	+ (26 y)	Proteinuria > 500 g/24 hours	UCCS (stopped)	
											Creatinine ↑		
28 y ♀	< 1y	Hypoglycemic seizures from birth	R83C	-	-	Osteopenia	+	+	+/+	+ (16 y)	-	CNGDF → UCCS	Brain damage with refractory epilepsy
													Polycystic ovaries
36 y ♀	3 y	Hypoglycemic seizures and hepatomegaly from 2 mo	Not performed	+ (corrected after liver transplantation)	+ (corrected after liver transplantation)	-	+	Epistaxis	-/-	+ (11 y)	-	CNGDF from 11 to 21 y → UCCS → Liver transplantation	Irregular mentruation cycles; Succesful pregnancy after liver transplantation
29 y ♂	2 mo	Hypoglycemia and hepatomegaly from 2 mo	Not performed	+++	+++	-	+	Epistaxis	-/-	+ (19 y)	Proteinuria > 400 mg/24 hours		

## Discussion

In the European Study on GSD type I (ESGSD I) almost 13% of the patients with GSD Ia presented before the age of 1 month and 50% presented between 1 and 6 months [1]. Fast-induced hypoglycemia and hyperlactacidemia may be the first symptoms in the neonatal period; more commonly, the presenting feature is a marked hepatomegaly around 3 to 6 months, which may be present at birth in some cases [2]. In our case series, hypoglycemic seizure in the neonatal period was the presenting symptom in the first and third cases, being hepatomegaly diagnosed when 5 months in the first case. Hypoglycemia and hepatomegaly presented at 2 months in cases 4 and 5. Exceptionally, the diagnosis was made in adulthood in one case by the presence of HCA. This patient didn’t have symptomatic hypoglycemia during childhood, but had hyperlactacidemia, which can be protective against cerebral symptoms of hypoglycemia, seeing that it may be used as an alternative fuel in cerebral metabolism [1, 2, 9].

The disturbed glucose homeostasis in GSD Ia can lead to secondary metabolic derangements, mainly hyperlactacidemia, hyperlipidemia and hyperuricemia.

Severe mixed hyperlipidemia and hepatic steatosis are present in most cases: elevated cholesterol was reported in 76% of cases and elevated triglycerides in 100%, in one study [11]. The underlying mechanisms responsible for hyperlypidemia are still largely unknown [12]. The marked hyperlipidemia may be due to increased *de novo* lipidogenesis and release of lipids into the blood compartment, owing to increased levels of acetyl coenzyme A [2, 8, 13], and decreased clearance and uptake, in account of reduced activity of lipoprotein lipase and hepatic lipase; so, lipolysis of circulation lipoproteins is impaired, leading to decreased triglyceride clearance. Also, the diminished uptake of low density lipoproteins (LDL) particles may contribute to the hypercholesterolemia often present [13]. Besides, the accumulation of G6P in the liver tissue may play a role through the activation of transcription of lipidogenic genes [8]. Some studies have demonstrated an increased production of very low density lipoproteins (VLDL), especially triglyceride-rich VLDL particles, related to the low concentration of insulin found in GSD type I patients, as insulin is known to suppress hepatic VLDL production [12–14]. This way, GSD type Ia patients have an increased number of high size (due to the accumulation of triglycerides) VLDL and LDL particles, associated with increased levels of ApoB100, and decreased high density lipoprotein (HDL) cholesterol and Apo A1 [13]. All of our male patients have marked hyperlipidemia, with raised Apo B100 levels. One of our female patients had mild hyperlipidemia, corrected after liver transplantation; opposed to most of the reports, our third case has normal lipid values, with a raised Apo A1 and HDL-cholesterol, which makes as suspect of presence of another metabolic defect, which counterbalances the hyperlipidemia frequently present in GSD Ia.

Despite marked hyperlipidemia since young ages, premature atherosclerosis [15, 16] has rarely been reported, unless associated with renal disease; protective factors [1, 12] may have a role, such as diminished platelet aggregation, hyperuricemia, increased levels of apolipoprotein E and decreased susceptibility of LDL to oxidation. However, a recent study involving 28 patients with GSD type I reported arterial dysfunction, with increased carotid intima media thickness, raising the possibility of increased cardiovascular risk in this group of patients [10]. This manifestation of subclinical atherosclerosis is present in our 2^nd^ case, in which, overt renal disease is absent.

Hyperlipidemia only partially responds to intensive dietary treatment, consisting of frequent meals, continuous nocturnal gastric drip feeding (CNGDF) or ingestion of slow-absorption carbohydrates [uncooked cornstarch (UCCS)] [5, 8, 17]. Recently, a novel dietary treatment, medium-chain triglycerides, which are absorbed via the portal vein without incorporation into chylomicrons, has demonstrated beneficial effects on blood triglycerides, lactic acid and glycemia, in a pilot study [18].

To reduce the risk of cholelithiasis and pancreatitis triglycerides lowering drugs are indicated; fibrates should be used, as they, through the activation of *PPARα*, increase lipoprotein lipase activity and have been shown to decrease triglyceride levels in this patients [12]; nicotinic acid can also be used [8]. Statins, which can lower plasma cholesterol levels, seems not to be indicated, unless progressive renal insufficiency occurs, since premature atherosclerosis is rare [8, 12]. However, more studies are needed to confirm or deny the presence of endothelial dysfunction in GSD type I patients. In what concerns fish oil supplements, they have been shown to improve hyperlipidemia in these patients [19], although with a modest effect (10 to 15% reduction of triglycerides compared with 50% achieved with fibrates) in one study [14]; however, the effect obtained isn’t long lasting and fish oil could lead to increased lipoprotein oxidation, augmenting atherogenicity [8, 15]; moreover, they reduce platelet aggregation and prolong bleeding time, and may exacerbate the bleeding tendency present in this disease [7]; so they are not indicated. In three of our patients fibrates were introduced; in one case it was associated with nicotinic acid with reduction of triglyceride levels of more than 60%; later, omega-3 fatty acids were introduced, with additional lipid reduction. In other case, fibrates were withdrawn, as they were associated with myosytis, although they were able to reduce triglyceride levels in about 70%; this patient has now started a statin, since he has end stage renal disease.

Hyperuricemia, reported to be present in 89% of the cases in one study [11], results from both increased production (decreased intra-hepatic phosphate concentration stimulates the degradation pathway of adenine nucleotides) and decreased renal clearance (competitive inhibition of uric acid excretion by lactate) [2, 8]. Complications due to hyperuricemia can occur, being urolithiasis and gouty arthritis the most frequent [1]. Despite this risk it might be advisable to keep uric acid concentration near the upper limit of normal range, since it is a potent radical scavenger [1, 8], with a possible protective role in the development of atherosclerosis. All but the third patient had hyperuricemia, with the need for treatment with xanthine oxidase inhibitor in 3 cases, one of them starting before 10 years old. In one case frequent gouty arthritis episodes were present. Notwithstanding treatment, they maintain elevated uric acid concentrations. On the other hand, the third case, despite the presence of other complications, has hypouricemia for unknown reason.

Several mutations have been identified through the coding and exon-intron junction regions of *G6PC*; in spite of not being more prevalent in any ethnic group, there are mutations that are unique to Caucasian, Hispanic, Asian and Jewish populations. Two of our cases have an identified mutation: in the third case, the missence mutation R83C in the exon 2 of the *G6PC* was found, one of the most frequent in the Caucasian population and known to completely abolish G6Pase-α enzyme activity [5, 6]. In the first case, the mutation found was IVS4-3C > G in the intron 4, which, till now, hasn’t been described in literature [3, 5, 6]; the functional tests and enzyme assays performed proved absence of G6Pase activity in this patient; analyzing all 5 cases this is probably the one with most severe disease expression. Until now a correlation between genotype and phenotype hasn’t been found [3, 5].

Two of our patients have borderline mental development (IQ 65–85), which is related to poor metabolic control, with frequent hypoglycemic episodes. In fact, altered brain function and structure, with abnormal electroencephalograms and magnetic resonance imaging (dilation of occipital horns and/or hyperintensity of subcortical white matter in the occipital or parietal lobes), might be associated with recurrent severe hypoglycemia [20]. Our third case presents brain damage and suffers from a refractory form of epilepsy, with crises not always related to hypoglycemic episodes. The other three cases have normal cognitive function.

GSD type Ia is characterized by growth retardation and delayed puberty, associated with poor metabolic control, particularly chronic metabolic acidosis, which interferes with growth hormone activity [21]; adequate dietary therapy has the ability to improve growth [2]. There is no place for growth hormone therapy nor testosterone or oestrogen supplementation, as they are not associated with improvement of the final height [8]. This could, in fact, be observed in the first two cases: despite testosterone supplementation to induce puberty, their final height wasn’t improved and both of them are short stature. On the other hand, in the fifth case, the introduction of CNGDF was associated with recovery of normal growth and both the fourth and fifth case are normal stature.

Besides hyperlipidemia and hepatomegaly, GSD type Ia patients usually present with anemia and increased bleeding tendency; anemia is reported in 81% of adult patients [11], and normally refractory to iron therapy; it may be associated with an up regulated production by HCA of hepcidin; this peptide hormone controls the intestinal iron absorption and release of iron from macrophages [2, 4, 8]. All of our cases had anemia, some since childhood before the presence of adenomas being detected. Unfortunately, hepcidin serum levels weren’t measured. Platelet dysfunction is common and recurrent epistaxis or gastrointestinal bleeding can be troublesome [1, 2, 8]. Four of our cases had frequent epistaxis; in one case recurrent episodes of upper gastrointestinal bleeding since childhood were associated with frequent hospital admissions and the need for exploratory laparotomy when 18 years old; later, adenomatous changes in the duodenal bulbous were identified as the source of bleeding; recently, a well differentiated neuroendocrine tumor of the duodenal wall was diagnosed, being at the moment under study.

Osteopenia, present in 55% of patients in a report involving 42 subjects with GSD type Ia [22], can be a risk factor for fractures later in life; it is thought to be a result of decreased bone matrix formation and decreased mineralization [8], probably in relation with muscle weakness, chronic acidosis and hypertriglyceridemia [23]; additionally, the hypercalciuria associated with hyperlactacidemia may contribute to the failure of normal bone accretion, since bone may have an important role as a buffer for the acid loads [24]. Beyond that, a lactose restricted diet may produce calcium depletion, making reasonable the use of calcium supplements in these patients [2, 8, 21, 24]. In one report, an association between low bone mineral density and low 25-hydroxy-vitamin D concentration was found [22], putting on scope the need for vitamin D supplementation. Since a high turnover can contribute to osteopenia, biphosphonates known to decrease bone turnover, may be of value in the treatment of osteoporosis in GSD type Ia patients [21]. Our first two cases have osteoporosis, one of them with significant bone mineral density gain after introduction of biphosphonates. The third case has osteopenia and the other two cases have normal bone mineral density.

The advances in dietary treatment resulted in longer survival rates and so, complications such as HCA and renal disease can occur.

HCA are usually detected between the 2^nd^ and 3^rd^ decades of life, with frequencies ranging from 16% to 75%, equal in both sexes [1, 25]. The mechanism responsible for their development remains unclear: perhaps a suboptimal metabolic control, with hypertriglyceridemia and hyperlactacidemia [25, 26], a hormonal imbalance between high glucagon and low insulin, or cellular glycogen overload [21] may play a role. Despite advancements in therapy, the incidence of adenoma formation remained largely unchanged over the past decades [25]; a reduction in size and/or number of adenomas has been observed in some patients under optimal metabolic control. HCA can produce symptoms related to mechanical compression or acute hemorrhage. Furthermore, albeit rare, malignant transformation into hepatocellular carcinomas can occur [1, 8, 27]. This implies that a regular follow up is needed: liver ultrasound once a year for the first 10 years; after that, once a year magnetic resonance imaging may be proposed. Should hepatic lesions be detected, the follow up must be intensified, with liver ultrasound or MRI every 3 to 6 months [2]. Serum α-fetoprotein and carcino-embryonal antigen can be used for malignant transformation screening, although false negatives can occur [8].

If recurrence of adenomas or increased risk for hepatocellular carcinoma exists, liver transplantation is a valid therapeutic option [4, 8], as long as metastatic hepatocellular carcinoma isn’t present [9]. Liver transplantation has the ability to correct metabolic defects, namely hypoglycemia, hyperlipidemia, hyperuricemia and growth retardation, improving the quality of life [28]. However, it doesn’t seem to have any effect on the prevention of renal failure [8, 29]. Combined liver-kidney transplantation can resolve both problems, and should be considered in GSD Ia patients with kidney dysfunction [30].

All of our patients have multiple, large liver adenomas; in case one, there was some regression in size and number of adenomas during follow up; on the other hand, in case 5 the number and size of adenomas raised despite optimization of dietary therapy during childhood. In the second case, the diagnosis was made during adulthood by the presence of HCA. Its recognition in adult age, if other manifestations can be found (in our patient hyperlactacidemia and growth retardation were present), should prompt the search for GSD type Ia [27]. In our fourth patient liver adenomas were recognized in the earliest age (11 years old), and she was transplanted when 27 years, with complete resolution of the metabolic imbalance. Renal disease wasn’t present before transplant and still there are no signs of renal involvement. In this case, liver transplantation was complicated by late acute cellular rejection, resolved after adjusting immunosuppressive therapy.

One of the most serious age related complications of GSD type Ia is renal disease, virtually present in all ineffectively treated patients [31]. Both glomerular and tubular functions may be affected. The first manifestation of glomerular disease is hyperfiltration with subsequent microalbuminuria, and then proteinuria and hypertension, which lately lead to renal failure, with a natural history similar to the renal disease in type 1 diabetes mellitus [1, 2]. The pathological findings include focal segmental sclerosis with interstitial fibrosis [21, 32, 33]. The etiology of renal involvement isn’t completely understood, but can be related to poor metabolic control [21], and the optimization of metabolic control can improve renal disease [34]. Some studies [4, 35, 36] have postulated a possible role of excessive renin-angiotensin activity in the development of hyperfiltration and progressive renal failure. When microalbuminuria is detected angiotensin converting enzyme inhibitors should be started without delay [37, 38]. Proximal tubular dysfunction (Fanconi like syndrome) seems to be related to poor metabolic control, being corrected after intensive dietary treatment [1, 8, 21, 31, 33, 39]. However, distal tubular dysfunction may appear even in patients with optimal metabolic control, and can lead to hypercalciuria and hypocitraturia, which pose a risk to kidney stone formation [40, 41]; supplementation with citrate may be of benefit in preventing or ameliorating urolithiasis and nephrocalcinosis. Regular kidney ultrasound is required [1, 8]. Hemodialysis and kidney transplantation may be necessary once end-stage renal disease is present [2, 8]. In our case report, two out of five have proteinuria without renal failure and not associated with nephrocalcinosis. Our first case has proteinuria since 6 years old, but angiotensin converting enzyme inhibitor was only introduced when 17. Progressive renal disease with nephrotic range proteinuria arose and this patient is now under regular hemodialysis program. Of notice is that one of the sibs of our 2^nd^ case has end-stage renal disease associated with focal segmental glomerulosclerosis also under hemodialysis.

Besides the commonly reported manifestations and complications, some conditions that might be associated with the disease are rarely recognized or reported. One of them is depressive illness requiring therapy; GSD type Ia poses an enormous burden for patients: a lifelong intensive dietary therapy for 24 hours and the fear for complications [1, 8]. In our report, both female patients had depressive illness needing anti-depressive medication.

Pulmonary hypertension is a very rare complication with poor prognosis reported in some patients. The etiology is unclear, but abnormalities in serotonin metabolism have been proposed [2, 4, 42, 43]. This complication hasn’t been reported in neither of our patients till now.

Although irregular menstruation cycles and polycystic ovaries are frequently described in GSD type Ia female patients, fertility seems not to be impaired, since there are reports of successful pregnancies [44, 45]. However, attention should be paid to the possibility of enlargement of HCA during pregnancy, so close monitoring is necessary [45]. Likewise, careful supervision in mandatory in the third trimester, as the rapid fetal growth in this period implies maintaining a state of euglycemia for achieving a normal fetal outcome [44]. One of our patients had a normal pregnancy after liver transplantation and one of our male patients has a healthy child.

## Conclusion

Since the first description of GSD I major progress has been made in what concerns the knowledge of the clinical, biochemical, genetic features and natural history of the disease. This allowed advances in therapy, mainly dietary therapy, with improvement of survival and quality of life. Hyperlipidemia, almost always present, should be treated to reduce the risk of cholelithiasis and pancreatitis. Increased cardiovascular risk, although rarely reported, is becoming a raising concern. In fact the frequent association of GSD I and renal dysfunction results in additional risk. Yet, the small number of patients in each center makes it difficult to have randomized clinical trials and the recommendations are based on clinical experience more than large scale studies.

## Consent

Written informed consent was obtained from the patient for publication of this Case Report and any accompanying images. A copy of the written consent is available for review by the Editor-in-Chief of this journal.

## Abbreviations

G6Pase-α: Glucose-6-phosphatase-α
GSD I: Glycogen storage disease type I
G6P: Glucose-6-phosphate
HCA: Hepatocellular adenomas
CNGD: Continuous nocturnal gastric drip feeding
UCCS: Uncooked cornstarch.
